# Single-Cell Transcriptomic Profile Associated with Sub-Subtype A6 and CRF63-02A6 HIV-1 Strain Infection

**DOI:** 10.3390/v18020204

**Published:** 2026-02-04

**Authors:** Kirill Elfimov, Anna Khozyainova, Ludmila Gotfrid, Dmitriy Baboshko, Dmitry Kapustin, Polina Achigecheva, Vasiliy Ekushov, Maksim Halikov, Mariya Gashnikova, Tatyana Bauer, Tatyana Tregubchak, Andrey Murzin, Arina Kiryakina, Aleksei Totmenin, Aleksandr Agaphonov, Natalya Gashnikova

**Affiliations:** 1 State Research Centre of Virology and Biotechnology ‘Vector’, Koltsovo 630559, Russia; 2 Cancer Research Institute, Tomsk National Research Medical Center, Russian Academy of Sciences, Tomsk 634009, Russia; 3 Novosibirsk Regional Clinical Infectious Diseases Hospital, Novosibirsk 630531, Russia; 4 Department of Natural Sciences, Novosibirsk State University, Novosibirsk 630090, Russia

**Keywords:** HIV-1, sub-subtype A6, CRF63_02A6, acute HIV infection, immune response, transcriptomics

## Abstract

We present the single-cell transcriptomic analysis of peripheral blood mononuclear cells (PBMC) from individuals during acute HIV-1 infection caused by viral strains circulating in Russia and the Former Soviet Union (FSU) countries. Using 10x Genomics single-cell RNA sequencing (scRNA-seq) on the Illumina NextSeq 550 platform, we have analyzed scRNA-seq data from three treatment-naive patients (viral load > 1 × 10^6^ copies/mL, estimated infection duration ≤ 4 weeks) and three healthy donors. Data integration (Seurat, Harmony), automated cell-type annotation (CellTypist), and GeneOntology (GO) enrichment analysis for highly expressed and low-expressed genes revealed a profound reorganization of transcriptional programs across key immune populations, including memory CD4^+^ and CD8^+^ T cells, non-classical monocytes and natural killer cells (NK-cells). We observed signatures of hyperactivation of pro-inflammatory pathways (NF-kB, TNF, and type I/II interferon signaling), upregulation of genes associated with cellular migration (*CXCR4*, *CCR7*) and metabolic adaptation (oxidative phosphorylation components), alongside a mixed pro- and anti-apoptotic expression profile. Notably, our data pointed to a pronounced dysregulation of the TGF-β and mTOR signaling cascades, disrupted intercellular communication networks—particularly between cytotoxic cells and their regulators—altered expression of genes implicated in disease progression (*OLR1*, *SERPINB2*, *COPS9*) and viral persistence control (*NEAT1*, *NAF1*). This work provides an initial single-cell transcriptional atlas characterizing early immune responses to HIV-1 sub-subtypes A6 and CRF63_02A6, the predominant drivers of the HIV epidemic across the FSU region.

## 1. Introduction

The acute phase of HIV-1 infection is marked by a sharp increase in viral load, depletion of CD4^+^ T cells, and a massive release of pro-inflammatory cytokines. Monocytes and dendritic cells (DCs) are among the first to sense HIV, leading to the production of interferons (IFN), IL-1, IL-2, and other signaling molecules [1]. This response triggers a “cytokine storm,” which often evolves into chronic inflammation in people living with HIV (PLWH), even during effective antiretroviral therapy (ART) with sustained viral suppression [2].

Notably, several signaling pathways involved in the antiviral response are exploited by the HIV-1 long terminal repeat (LTR) to enhance viral gene transcription. A key example is the TNF-α signaling cascade, which activates the NF-kB pathway. NF-kB then binds to its cognate sites within the HIV-1 LTR [3]. This binding potently activates viral transcription, as the 5′ LTR region contains three binding sites for the transcription factor Sp1, which is integral to this NF-kB-dependent activation [4].

In the absence of ART, HIV-1 infection leads to massive immune activation and apoptosis of CD4^+^ T lymphocytes, a process markedly accelerated during the acute phase [1]. Apoptotic cell death is driven both by intracellular signaling cascades, such as Fas/FasL [5], and by cytotoxic effector cells—including cytotoxic T lymphocytes (CTLs), CD8^+^ effector memory T (CD8^+^ Tem) cells, and NK cells—which identify and eliminate infected targets through MHC-I recognition [6]. Concurrent with heightened cytotoxic activity, the proportion of antiviral mononuclear subsets—including non-classical monocytes, NK cells, and CD8^+^ central (CD8^+^ Tcm) and CD8^+^ Tem—increases [7]. Another key process is the establishment of a diverse proviral reservoir, which includes a latent fraction. During infection, immune cells actively upregulate genes associated with tissue homing (e.g., CXCR4, CD62L, CD38, CCL2). These migrating infected cells then disseminate HIV to lymphoid tissues (including lymph nodes, spleen, and gut-associated lymphoid tissue), the central nervous system (CNS), and other anatomical sanctuaries [8,9]. Furthermore, host factors that suppress viral transcription, such as NAF1, contribute to the formation of this latent reservoir by effectively pausing the viral life cycle without eliminating the silent provirus [10].

It is well established that different Human Immunodeficiency Virus (HIV) subtypes and circulating recombinant forms (CRFs) exhibit distinct host–pathogen interactions. For instance, subtype C strains isolated in Uganda and Zimbabwe demonstrate slower disease progression rates compared to co-circulating subtypes A and D from the same region [11]. In the Russian Federation, HIV-1 sub-subtype A6 and CRF63_02A6 have become the predominant circulating variants [12]. Nevertheless, these strains remain less thoroughly characterized than more globally prevalent subtypes, such as subtype B.

In recent years, omics technologies, particularly transcriptomics, have become integral to HIV research. A transformative advancement in this field is single-cell RNA sequencing (scRNA-seq), which enables the detailed dissection of antiviral immune responses within specific cell populations. scRNA-seq has already been employed in studies focusing on the acute phase of HIV-1 infection [13,14]. However, individual and ethnic variations underscore the necessity for further research to build representative datasets. Moreover, single-cell transcriptomic data from HIV-positive individuals infected with HIV-1 sub-subtype A6 and CRF63_02A6—the dominant strains circulating in Former Soviet Union (FSU) countries are still lacking.

The introduction of sub-subtype A6 into the Soviet Union is historically linked to the period of political cooperation with African nations in the 1970s, with migration flows (students, specialists, military personnel) serving as a potential transmission route [15]. Initially spreading among people who inject drugs (PWID), HIV-1 sub-subtype A6—the primary driver of the Russian epidemic—has since shifted to predominantly sexual transmission and now dominates across large parts of the FSU. CRF63_02A6 is a recombinant form derived from sub-subtype A6 and the Central Asian CRF02_AG. Phylodynamic analysis indicates that this virus most likely emerged in Novosibirsk, Russia, between 2004 and 2005 and has since disseminated widely across various regions of Russia and Central Asian countries [16].

This study aims to address the existing knowledge gap in the single-cell transcriptomic landscape of peripheral blood mononuclear cells (PBMCs), with a specific focus on key antiviral cell populations, including CD4^+^ T lymphocytes, CD8^+^ T lymphocytes, monocytes, DCs, NK cells, and B lymphocytes. We sought to fill the critical void of transcriptomic data for people living with HIV (PLWH) infected with HIV-1 strains circulating in the FSU region. Our work encompasses viral isolate genotyping, a comprehensive overview of the global transcriptional profile, a detailed characterization of antiviral mononuclear cell populations, a comparative analysis of activated and suppressed signaling pathways against a study control cohort, and an in-depth investigation of cell–cell communication networks.

## 2. Materials and Methods

### 2.1. Study Cohorts

This study enrolled HIV-positive individuals and healthy donors (confirmed negative for HIV antigens and antibodies) aged 20 to 50 years (Supplementary Table S1). All participants provided written informed consent for cytological and molecular genetic testing, as well as for the analysis and storage of the resulting data. The study protocol was approved by the Ethics Committee of the State Budgetary Healthcare Institution “Kraevaya Klinicheskaya Bolnitsa No. 2” (Regional Clinical Hospital No. 2) in Vladivostok (Extract from Meeting Protocol No. 31/1, dated 10 January 2024).

Inclusion criteria for HIV-positive patients in this study were as follows: (I) an estimated duration of infection of ≤4 weeks prior to blood draw (patients experienced an event that they associate with the risk of contracting HIV during this time.); (II) negative or indeterminate HIV-1 Western blot results; (III) plasma viral load ≥ 1 × 10^6^ HIV RNA copies/mL (Fiebig stages I–IV); and (IV) the availability of self-reported data on ethnicity, presumed transmission route, medical history, and any concurrent secondary diseases. Patients were also classified according to the Pokrovsky staging system, which is the standard clinical classification for HIV infection used in Russia [17]. Within the study’s HIV-positive cohort, individuals were classified at the time of blood sampling as stage 2B (acute stage without secondary diseases; patient YAK) and stage 2C (acute stage with transient secondary diseases; patients KOD and SMA). A detailed description of this staging system is provided in Supplementary Table S1, ‘Clinically relevant information for HIV-positive patients’. Whole-blood biospecimens (9–10 mL) were transported to the laboratory within 4 h of collection. PBMCs were isolated within 8 h post-draw, cryopreserved, and subsequently stored in the vapor phase of liquid nitrogen (−196 °C) for long-term biobanking.

For the control samples, this study utilized single-cell transcriptomic data from a previous study investigating chromatin landscape remodeling in peripheral blood cells from patients with severe infection from the Delta variant of COVID-19 (NCBI BioProject accession number: PRJNA1164162) [18]. We used PBMC transcriptome sequencing data (raw reads) from healthy people (without COVID-19) from this study (BioSample accession numbers: SAMN43886591, SAMN43886592, SAMN43886594), which matched our patients by gender, age range, and ethnicity (Supplementary Table S1 ‘Clinically relevant information for HIV-positive patients’). The last important criterion for selecting people from the control cohort of the study was a set of sample preparation for single-cell sequencing, which was similar to the one used in this study Next GEM Single Cell 3′ GEM Kit v3.1 (10x Genomics, Pleasanton, CA, USA; Cat. No. PN-1000128).

### 2.2. Nucleic Acid Extraction and PCR for HIV-1 Whole Genome Sequencing

Total RNA was extracted from peripheral blood plasma using a column-based method with the “RNA Extraction Kit from Blood Plasma, diaGene” (diaGene, Moscow, Russia; Cat. No. 3324.0250). All procedures were performed in strict accordance with the manufacturer’s protocol. The isolated RNA was stored at −70 °C until further use.

### 2.3. PCR for HIV-1 Near-Full-Length Genome Amplification

Near-full-length genome (NFLG) sequences of HIV-1 were obtained using a two-step nested PCR approach with custom-designed (in-house) primers. cDNA synthesis was performed using the “BioMaster RT-PCR-Extra” reagent kit (BioLabMix, Novosibirsk, Russia; Cat. No. RM06-200). The second-round PCR amplification was carried out using the “BioMaster LR HS-PCR” long-range PCR kit (BioLabMix, Novosibirsk, Russia; Cat. No. MH040-400). End-point PCR products were visualized by electrophoresis on a 0.75% agarose gel stained with ethidium bromide.

The resulting PCR amplicons were purified using a column-based cleanup method with the Cleanup S-Cap kit (Eurogen, Moscow, Russia; Cat. No. BC041L), following the manufacturer’s instructions. Following purification, the virus-specific fragments were sequenced on an Illumina MiSeq platform (Illumina, Inc., San Diego, CA, USA; Cat. No. SY-410-1003).

### 2.4. HIV-1 Isolate Genotyping

Viral genome assembly was performed using the software packages BWA v.0.7.17 and iVar v.1.2.2. The resulting HIV-1 sequences were aligned against reference sequences of various subtypes and recombinant forms from the international GenBank database using MEGA11 and AliView [19,20]. Multiple sequence alignment was conducted with MAFFT v.7.526 (RIMD) under default parameters [21]. A maximum likelihood phylogenetic tree was constructed using the IQ-TREE web server v.2.4.0 with 1000 bootstrap replicates under the GTR + I + G substitution model, and topology robustness was assessed via bootstrap analysis [22]. The phylogenetic tree was visualized using the Interactive Tree of Life toolkit [23].

The recombination analysis was performed using RIP 3.0 software [24] in the HIV sequence database (https://www.hiv.lanl.gov/content/sequence/RIP/RIP.html (accessed on 1 December 2025)).

### 2.5. Isolation, Cryopreservation, and Thawing of Cells

PBMCs were isolated from whole blood samples using a Ficoll-1077 density gradient (Dia M, Moscow, Russia; Cat. No. Diacoll 1077), following the 10x Genomics protocol “Isolation of Leukocytes, Bone Marrow and Peripheral Blood Mononuclear Cells for Single Cell RNA Sequencing”. Briefly, whole blood was diluted 1:1 with 1× PBS (Gibco, Waltham, MA, USA; Cat. No. 10010023), and 10 mL of diluted blood was layered over 5 mL of Ficoll-1077 (Dia M, Moscow, Russia; Cat. No. Diacoll 1077) in a 15 mL conical tube (Corning, Corning, NY, USA; Cat. No. 430053). Centrifugation was performed at 300 rcf for 30 min (modified from the original 10x Genomics protocol). The intermediate white ring containing mononuclear cells was carefully collected into a new 15 mL conical tube (Corning, Corning, NY, USA; Cat. No. 430053).

Following isolation, PBMCs were washed with 10 mL of RPMI-1640 culture medium (Gibco, Waltham, MA, USA; Cat. No. 11875093) by centrifugation at 300 rcf for 10 min. Cell counting was performed using a TC20 Automated Cell Counter (Bio-Rad, Hercules, CA, USA; Cat. No. 1450102). A second wash with RPMI-1640 medium (Gibco, Waltham, MA, USA; Cat. No. 10010023) was conducted under identical conditions. After the second wash, PBMC were resuspended in Fetal Bovine Serum (Gibco, Waltham, MA, USA; Cat. No. A5256701) to a final concentration of 6 × 10^6^ cells/mL. The cell suspension was aliquoted (1 mL per vial) into pre-chilled cryovials (Corning, Corning, NY, USA; Cat. No. 431386), each containing 1 mL of freezing medium (30% DMSO (Servicebio, Wuhan, China; Cat. No. G5051-100ML), 70% FBS (Gibco, Waltham, MA, USA; Cat. No. A5256701)).

Cryovials containing the cell suspension in freezing medium were transferred to a CoolCell SV2 freezing container (Corning, Corning, NY, USA; Cat. No. BCS-172) and placed at −80 °C for 24 h. After this period, vials were rapidly transferred (within 1–2 min) to liquid nitrogen (−196 °C) for long-term storage (1–3 months) to prevent thawing.

Cell thawing prior to single-cell RNA sequencing library preparation was performed according to the 10x Genomics protocol "Fresh Frozen Human Peripheral Blood Mononuclear Cells for Single Cell RNA Sequencing". Specifically, cryovials were transported from storage to a biosafety cabinet using the CoolCell SV2 container (Corning, Corning, NY, USA; Cat. No. BCS-172) and thawed in a 37 °C water bath for approximately 2 min. Cells were washed with 1× PBS (Gibco, Waltham, MA, USA; Cat. No. 10010023) by centrifugation at 300 rcf for 5 min and resuspended in 200 μL of 1× PBS (Gibco, Waltham, MA, USA; Cat. No. 10010023). A 10 μL aliquot of cell suspension was mixed with 10 μL of 0.4% Trypan Blue (Abisense, Moscow, Russia; Cat. No. DYE-01-4-100ML), and cell count/viability was assessed using the TC20 Automated Cell Counter (Bio-Rad, Hercules, CA, USA; Cat. No. 1450102).

### 2.6. Preparation and Sequencing of PBMC Single-Cell RNA Libraries

Single-cell emulsions were generated using the Chromium Controller (10x Genomics, Pleasanton, CA, USA; Cat. No. PN 120270) with a chip from the Chromium Next GEM Chip G Single Cell Kit (10x Genomics, Pleasanton, CA, USA; Cat. No. PN-1000127) and beads from the Next GEM Single Cell 3′ v3.1 Gel Beads kit (10x Genomics, Pleasanton, CA, USA; Cat. No. 1000128). Reverse transcription, cDNA amplification, and cleanup were performed using the Next GEM Single Cell 3′ GEM Kit v3.1 (10x Genomics, Pleasanton, CA, USA; Cat. No. PN-1000128). Buffer EB (Qiagen, Hilden, Germany; Cat. No.19086) was used for elution during magnetic bead-based cDNA purification, and freshly prepared 80% ethanol was used for washing. cDNA amplification was performed for 12 cycles, as recommended by the manufacturer’s protocol based on the estimated cell count.

Prior to library preparation, cDNA quality was assessed. Fragment length distribution was analyzed using the TapeStation 4200 system (Agilent Technologies, Santa Clara, CA, USA; Cat. No. G2991BA) with High Sensitivity D5000 ScreenTapes (Agilent Technologies, Santa Clara, CA, USA; Cat. No. 5067-5592), while cDNA concentration was quantified using the Qubit 3.0 Fluorometer (Thermo Fisher Scientific, Waltham, MA, USA) with the Qubit dsDNA HS Assay Kit (Thermo Fisher Scientific, Waltham, MA, USA).

Libraries were constructed using the Library Construction Kit (10x Genomics, Pleasanton, CA, USA) and Next GEM Single Cell 3′ GEM Kit v3.1 (10x Genomics, Pleasanton, CA, USA), with sample indexing accomplished using the Dual Index Kit TT Set A (10x Genomics, Pleasanton, CA, USA). Indexing PCR was performed for 12 cycles using cDNA input concentrations ranging from 4 to 25 ng/μL.

Final library quality control was performed using the TapeStation 4200 system (Agilent Technologies, Santa Clara, CA, USA) with High Sensitivity D5000 ScreenTapes and the Qubit 3.0 Fluorometer (Thermo Fisher Scientific, Waltham, MA, USA; Cat. No. Q33216) with the Qubit dsDNA HS Assay Kit (Thermo Fisher Scientific, Waltham, MA, USA; Cat. No. Q32854).

Sequencing was performed on a NextSeq 550 instrument (Illumina, Inc., San Diego, CA, USA, Cat. No. SY-415-1002) using the NextSeq 550 High-Output Kit (Illumina, Inc., San Diego, CA, USA; Cat. No. 20024908) (300 cycles) for paired-end sequencing (2 × 150 bp).

All steps for cDNA and library preparation were conducted according to the manufacturer’s protocols.

### 2.7. Single-Cell RNA-Seq Data Preprocessing and Annotation

Raw sequencing data were processed using CellRanger (8.0.1) for alignment and generation of gene-cell count matrices. Quality control and downstream analyses were performed in Seurat (5.2.0) [25]. Multiplets were identified and removed using scDblFinder (1.14.0) with an expected doublet rate of 0.8% per 1000 cells [26]. Cells expressing fewer than 200 detected genes or fewer than 600 UMIs were excluded. For samples from individuals with HIV infection, cells with >17.5% mitochondrial gene content were removed. These criteria for cell exclusion (filtration) were selected based on similar criteria for human cells in the control cohort of the study. scRNAseq data for the study’s control cohort were taken from an open source of another study [18].

Sequencing quality control involved assessment of key metrics generated by Cell Ranger v.8.0.1 during the cellranger count pipeline execution, provided in the web_summary.html report. These metrics included reads per cell, genes detected per cell, valid barcodes and UMIs, UMIs per cell, sequencing saturation, Q30 scores for RNA reads, and Q30 scores for UMI reads. The sequencing data met all standard quality control metrics for PBMC single-cell RNA-seq. Specifically, the median sequencing depth exceeded 40,000 reads per cell, with medians of ~2000 genes and >2500 UMIs detected per cell.

Normalization and variance stabilization were performed using SCTransform v2. The top 30 principal components (PCs) were integrated across samples and corrected for batch effects using Harmony. Harmony-adjusted PCs were used for construction of the shared nearest-neighbor (SNN) graph, uniform manifold approximation and projection (UMAP) embedding, and Louvain clustering using default parameters.

Normalization and variance stabilization were performed using SCTransform v2. Principal component analysis (PCA) was conducted on the SCT assay using 50 components. Batch correction and dataset integration were performed with Harmony (1.2.3) us-ing the sample identity as the batch variable (group.by.vars = “sample”). Harmony was run for 20 iterations, and the first 30 Harmony-adjusted principal components were used for downstream analyses.

Harmony-corrected embeddings were used to construct the shared nearest-neighbor (SNN) graph, followed by Louvain clustering (resolution = 0.8) and uniform manifold ap-proximation and projection (UMAP) visualization. Integration quality was assessed by visual inspection of UMAP embeddings colored by sample and condition.

Differential expression analysis was performed using the FindAllMarkers function on the RNA assay after LogNormalize normalization. Cell type annotation combined manual and automated approaches. Manual annotation was based on cluster-specific differentially expressed genes and canonical marker expression (Supplementary Table S2). Automated annotation employed the Azimuth reference-based mapping workflow using the PBMC reference dataset [27]. Clusters 11 and 14 were manually identified as B cells, after which the corresponding B-cell subtype annotations were assigned according to the Azimuth reference predictions. Likewise, mucosal-associated invariant T-cell (MAIT), gamma-delta T cell (γδT cell), and double negative T cell (dnT cell) labels were assigned according to the Azimuth reference. Clusters 17, 19, 20, and 26 were excluded from downstream analyses due to low quality.

To evaluate the efficacy of this correction and the robustness of our clustering, we conducted a donor-level sensitivity analysis. We visually inspected the distribution of cells from each individual donor before and after integration (Supplementary Figure S2, ‘UMAP graph before integration’, and Supplementary Figure S3, ‘UMAP graph after integration’). Furthermore, to explicitly test whether our key findings were driven by a single outlier individual, we generated UMAP embeddings and highlighted the clustering analysis for each donor separately (Supplementary Figure S4, ‘UMAP graph for separate donors’). The consistent distribution of major cell populations across all individual plots confirmed that no single donor disproportionately influenced the integrated cluster structure or the overall transcriptional landscape.

### 2.8. Pseudobulk Differential Expression Analysis

Pseudobulk differential expression analysis was performed at two levels. First, a global immune-cell-level pseudobulk comparison was conducted by aggregating all immune cell populations, excluding Plasmablasts/Plasma cells, Platelets, and Proliferating T/NK cells. Second, differential expression was assessed at the level of individual cell types, comparing patients with HIV and controls within each annotated population. Differential expression was computed using the FindMarkers function with the DESeq2 method. Volcano plots were generated using EnhancedVolcano (GitHub URL: https://github.com/kevinblighe/EnhancedVolcano; access date: 14 January 2026) [28]. Gene Ontology (GO) pathway enrichment analyses were performed with clusterProfiler (4.12.6) [29], using genes with adjusted *p*-value < 0.05 and log2fold change > 0.5.

Heatmaps of global immune pseudobulk results were generated using the pheatmap package (1.0.12) and constructed from the top 25 upregulated and top 25 downregulated genes (adjusted *p*-value < 0.05), ranked by log2 fold change, and based on scaled pseudo-bulk expression values across individual donors (https://github.com/raivokolde/pheatmap (accessed on 1 December 2025)). Descriptive statistics, including mean expression, standard deviation, and 95% confidence intervals, were calculated using log-normalized pseudobulk expression values and are reported in the Supplementary Tables (Supplementary Table S5, ‘Results of pseudobulk RNAseq analysis’).

Stability of the global immune pseudobulk analysis was evaluated by repeating the analysis on a random subsample comprising 70% of cells from both HIV-positive and control groups. Corresponding differential expression tables and descriptive statistics are provided as Supplementary Material (Supplementary Table S3, ‘Results of the validation pseudobulk differential expression analysis (70% subsampling)’; Supplementary Table S4, ‘The level of gene expression within the study groups’).

### 2.9. Inference of Cell–Cell Communication Networks

Cell–cell communication was inferred using the CellChat package (2.1.2) [30]. Each dataset was processed independently following the “Inference and analysis of cell–cell communication using CellChat” vignette. Ligand–receptor interactions were computed using the standard workflow, and interactions were filtered with filterCommunication (min.cells = 10, min.samples = 3). Comparative analysis across conditions was performed according to the “Comparison analysis of multiple datasets using CellChat” vignette, allowing direct comparison of communication networks across datasets with matched cell-type composition.

## 3. Results

### 3.1. Genetic Diversity of HIV Isolates

**Figure 1:**
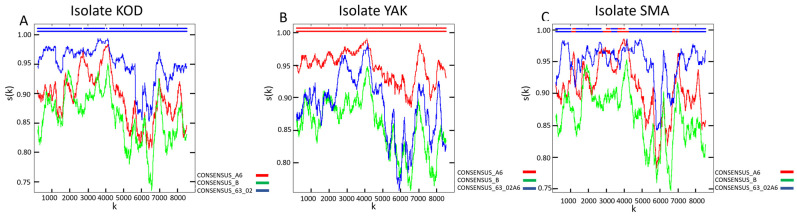
Recombination analysis of HIV-1 strains isolated from the biological material of the study’s HIV-positive cohort. The analysis was performed using RIP 3.0 software [24] on the web resource of the HIV sequence database (https://www.hiv.lanl.gov/content/sequence/RIP/RIP.html (accessed on 1 December 2025)): (**A**) Nucleotide sequence homology of isolate KOD compared to the reference nucleotide sequences of sub-subtype A6, subtype B, and CRF63_02A6. (**B**) Nucleotide sequence homology of isolate YAK compared to the reference nucleotide sequences of sub-subtype A6, subtype B, and CRF63_02A6. (**C**) Nucleotide sequence homology of isolate SMA compared to the reference nucleotide sequences of sub-subtype A6, subtype B, and CRF63_02A6. This spectrum of variants allows for the characterization of the single-cell transcriptomic landscape of HIV-1 infection caused by strains that are prevalent across FSU nations but remain less studied compared to globally dominant subtypes and CRFs.

Genotyping identified HIV-1 genetic variants belonging to CRF63_02A6 (patient KOD; GenBank accession number: PX653452), sub-subtype A6 (patient YAK; GenBank accession number: PX653454), and a unique recombinant form (URF) classified as URF_63/A6 (patient SMA; GenBank accession number: PX653453) in the study cohort (Figure 1). CRF63_02A6 and sub-subtype A6 are among the most prevalent HIV-1 genetic variants in Russia and other FSU countries [12]. The identified URF is primarily composed of a CRF63_02A6 backbone with interspersed fragments of sub-subtype A6.

### 3.2. Single-Cell Transcriptomic Profiling

**Figure 2:**
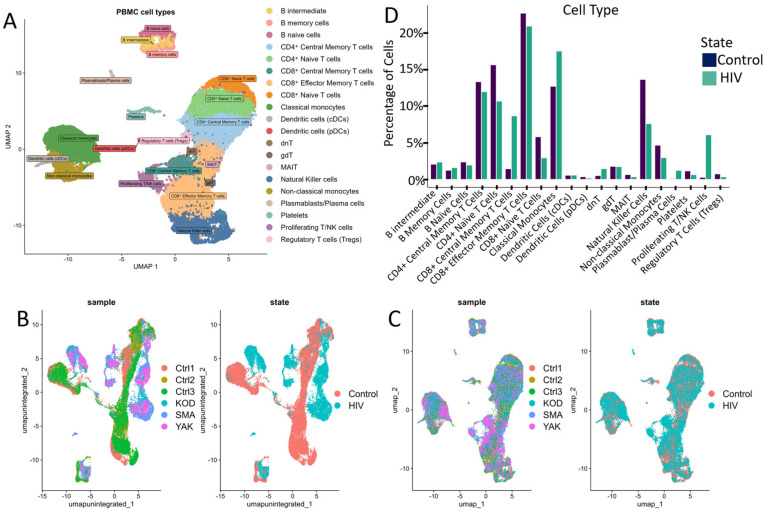
Cell cluster analysis: (**A**) UMAP graph of all cells after integration with a unique color designation for each annotated cluster. (**B**) UMAP visualization of cells before integration with a unique color for each patient. (**C**) UMAP visualization of cells after integration with separate colors for HIV status (graph on the right). (**D**) Proportion of cells in individual PBMCs populations from the total number of analyzed PBMCs for HIV-positive and HIV-negative people. When comparing the proportions, no statistically significant differences were found after the Bonferroni correction.

This study sequenced the transcriptomes of 13,845 single cells from three HIV-positive individuals during the acute stage of infection. As a control, we utilized transcriptomic data from PBMCs of 3 healthy donors obtained from a study investigating chromatin remodeling during COVID-19 (Figure 2B,C) [18]. Both datasets were generated using the same 3′ expression assay—Next GEM Single Cell 3′ Reagent Kits v3.1 protocol (10× Genomics, USA). We selected three healthy patients from this study based on their gender, age, and ethnicity (Supplementary Table S1 ‘Clinically relevant information for HIV-positive patients’). According to the published study, cell sample preparation, sequencing and filtration of low-quality cells were carried out in a similar way.

Following data integration and clustering, we annotated cell populations (Figure 2A) through label transfer using curated markers from comparable studies and reference databases (Supplementary Table S2).

The annotated clusters encompassed all major PBMC populations, including key adaptive and innate immune cell subsets: CD4^+^ and CD8^+^ T cell subsets (naive, central memory, effector memory), natural killer (NK) cells, dendritic cells (DCs), monocyte subsets (classical, intermediate, non-classical), B cells, and regulatory populations (regulatory T cells, γδT cells, dnT cells, MAIT cells).

Quantitative analysis of the cellular composition of PBMCs revealed notable (but not significant after Bonferroni correction; *p* > 0.001) changes in the percentage of mononuclear cell subpopulations between HIV+ and control cohorts (Figure 2D). Our data indicated a decreased proportion of naive CD4^+^ and CD8^+^ T cells (Figure 2D), consistent with antigen-driven differentiation and potential direct virus-mediated cytopathic effects on CD4^+^ T cells. This trend is consistent with the high plasma viral loads (>10^6^ HIV RNA copies/mL) measured in all patients from the HIV^+^ cohort (Supplementary Table S1). Concurrently, we noted an expansion of CD8^+^ effector memory T cells and Natural Killer cells (NK-cells), indicative of an active antiviral cytotoxic response. Notably, in our cohort of three HIV-positive individuals, we observed an expansion of proliferating T/NK cells alongside a marked reduction in regulatory T cells (Tregs) and mucosal-associated invariant T (MAIT) cells, suggesting a state of generalized immune activation.

**Figure 3:**
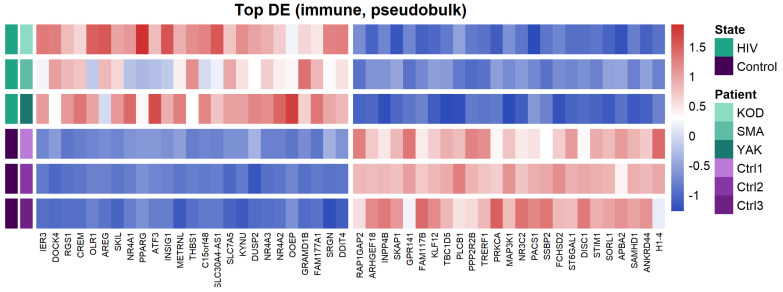
Heatmap of the top 50 differentially expressed genes (DEGs) between PLWH and HIV-negative patients. Distinct expression patterns between HIV-positive and control cohorts, identified by pseudobulk analysis of PBMC transcriptomes.

To characterize the overall transcriptomic profile of PBMCs, we performed a pseudobulk differential expression analysis (Figure 3).

Pseudobulk analysis of HIV-positive samples revealed upregulation of genes associated with immune activation (*NR4A1*, *NR4A2*, *NR4A3*), negative regulation of signaling (DUSP1, DUSP2, RGS1), pro-inflammatory responses (*THBS1*, *SRGN*, *OLR1*), and cellular stress (*ATF3*, *IER3*). This transcriptional profile indicates a dysregulation of cellular signaling and the onset of chronic inflammation, which is characteristic of HIV infection.

Among the downregulated genes, we identified those regulating cytoskeletal organization and cell migration (*ARHGEF2*, *ARHGEF18*), G-protein and purinergic receptor signaling (*P2RY8*, *GPR141*), as well as sphingolipid metabolism (*SMPD1*). Notably, we also observed the suppression of these regulatory molecules alongside the reduced expression of the transcription factor PPARG.

### 3.3. CD4^+^ Central Memory T-Cells (CD4^+^ Tcm)

During HIV infection, Tcm cells partially compensate for the depletion of effector CD4^+^ T cells through proliferation, helping to maintain the CD4^+^ T cell pool [31]. However, their longevity and proliferative capacity also make them a key cellular and latent reservoir for HIV, contributing to viral persistence during antiretroviral therapy [32]. During acute HIV infection, Tcm cells represent a primary viral target due to their high surface expression density of the CCR5 co-receptor [33].

**Figure 4:**
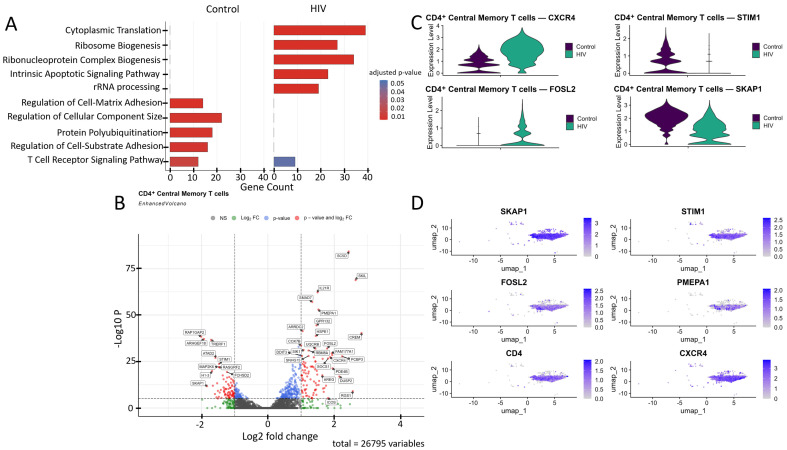
Transcriptomic profile of CD4^+^ Tcm cells. (**A**) Gene Ontology (GO) enrichment analysis for control (left) and HIV-positive (right) cohorts. (**B**) A volcano-plot demonstrating highly and lowly expressed genes in the CD4^+^ Tcm cells of HIV-positive cohort showing statistically significant differential expression compared to the control cohort. (**C**) Expression levels of key genes across cohorts. (**D**) Expression levels of key genes within the HIV-positive CD4^+^ Tcm cluster.

Comparative Gene Ontology (GO) enrichment analysis (Figure 4A) in CD4^+^ Tcm cells suggested that healthy donors exhibit a predominance of biological processes related to cell adhesion, cytoskeleton organization, and signaling (regulation of cell–matrix adhesion, actin filament organization, focal adhesion assembly, T cell receptor signaling pathway), likely reflecting their preparedness for migration, intercellular interactions, and immune surveillance. In contrast, cells from HIV-positive individuals in our cohort showed transcriptional patterns indicative of upregulated genes involved in metabolic processes (cytoplasmic translation, oxidative phosphorylation, ATP synthesis, electron transport chain), suggesting an activated cell state with increased energy demands.

CD4^+^ Tcm cells exhibited a statistically significant (*p* < 0.001) upregulation of genes including *IL21R*, *ICOS*, *SOCS1*, *CXCR4*, and *RGS1* (involved in immune response and T-cell signaling); *SKIL*, *SMAD7*, and *PMEPA1* (related to the TGF-β signaling pathway and its regulation); *DDIT3*, *FOSL2*, and *SIK1* (associated with cellular stress induction and apoptosis); *DUSP2*, *PDE4B*, *CREM*, *RBM8A*, *GPR132*, and *FAM177A1* (participants in various signaling pathways—phosphatases, phosphodiesterases, transcription factors, and other proteins); *COX7B*, *UQRCB*, and *HSPE1* (involved in energy metabolism and ATP generation); *SC5D* (cholesterol biosynthesis); *AREG* (epithelial cell regeneration and proliferation); *PCBP3* (mRNA stabilization); and *ARRDC2* (poorly characterized functions), alongside a downregulation of the genes *RAP1GAP2*, *TRERF1*, *ARHGEF18*, *ATAD2*, *STIM1*, *MAP2K6*, *RASGRF2*, *H1-3*, *SKAP1*, and *FCHSD2* (Figure 4B).

We focused on several key dysregulated genes with potential significance for HIV pathogenesis (Figure 4C,D). Upregulation of TGF-β pathway negative regulators (*SMAD7*, *PMEPA1*, *SKIL*) may have a dual role in pathogenesis. Although the suppression of the TGF-β pathway via the products of these genes may reduce the number of activated CCR5^+^ CD4^+^ T cells susceptible to HIV-1 [34], it also weakens TGF-β-mediated apoptosis of infected cells, potentially contributing to the maintenance of viremia. Upregulation of *CXCR4* and *FOSL2* may further promote viral persistence. The existence of dual-tropic (R5/X4) and CXCR4-tropic HIV strains expands the range of target cells through the use of the CXCR4 co-receptor [35], while the hyperexpression of *FOSL2*, a negative regulator of Th17 differentiation, may increase the vulnerability of mucosal surfaces to opportunistic infections by disrupting the expression of mucosal defense genes [36]. Reduced *STIM1* expression impairs calcium signaling and weakens the antigen response [37]. Finally, downregulation of proliferation-associated genes (*SKAP1*, *RASGRF2*) may limit clonal expansion of CD4^+^ T cells [38,39], depleting the lymphocyte pool and exacerbating immune deficiency.

### 3.4. CD8^+^ T-Central Memory Cells (CD8^+^ Tcm)

During the acute phase of HIV-1 infection, CD8^+^ Tcm cells play a critical role in controlling viremia. The presence of HIV triggers a rapid expansion in the diversity and quantity of CD8^+^ T cells, including Tcm precursors. As early as 3–7 days post-infection, a 2.7-fold increase in stem-like memory T cells (Tscm) is observed [40]. Notably, earlier activation of naive CD8^+^ T cells is associated with a lower viral set point and, likely, a smaller future proviral reservoir [41].

**Figure 5:**
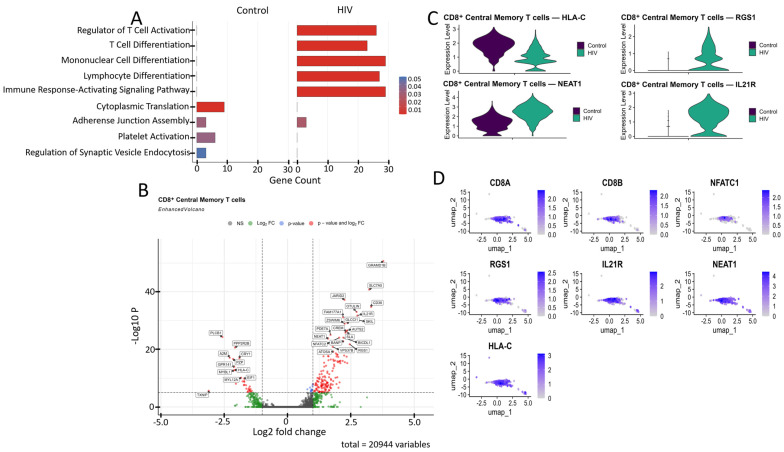
Characterization of the transcriptomic profile of CD8^+^ Tcm cells: (**A**) Gene Ontology (GO) annotation of expressed genes in the control and study cohorts. (**B**) A volcano-plot demonstrating highly and lowly expressed genes in the CD8^+^ Tcm cells of HIV-positive cohort showing statistically significant differential expression compared to the control cohort. (**C**) Expression levels of the most significant genes in the HIV-positive and control cohorts. (**D**) Expression levels of the most significant genes within the CD8^+^ Tcm cell cluster of HIV-positive individuals.

Comparative GO annotation analysis revealed that healthy individuals exhibit a predominance of pathways associated with fundamental cellular processes, such as cytoplasmic translation and adhesive junction assembly, reflecting homeostatic maintenance under normal conditions. In contrast, the acute phase of HIV infection is characterized by a pronounced shift towards an adaptive immune response, as evidenced by a significant enrichment of pathways regulating T cell activation and differentiation, as well as signaling mediated by surface immune receptors (Figure 5A).

CD8^+^ Tcm cells from HIV-positive individuals demonstrated upregulated expression of several genes compared to the study’s control cohort (Figure 5B), including: *CD38*, *IL21R*, *NFATC2*, *RGS1*, and *SLA* (regulation of immune response and T-cell signaling); *JARID2*, *AUTS2*, *BANP*, *ZSWIM6*, and *ATOSA* (epigenetic regulation and transcriptional control); *SLC7A5*, *PDE7A*, *CREM*, *SKIL*, *OTULIN*, and *FAM177A1* (intracellular signaling); *NEAT1* (post-transcriptional regulation); *GLCCI1* (apoptosis regulation); and *GRAMD1B*, *BICDL1*, and *VPS37B* (intracellular transport). The list of downregulated genes relative to the control group included: *PLCB1*, *PPP2R2B*, *TXNIP*, and *GPR141* (signal transduction regulation); *MYBL1* (cell cycle and replication control); *HLA-C* (antigen presentation via MHC-I); *CRY1* (circadian rhythm regulation); *A2M* and *PZP* (protease and cytokine binding); and *MYL12A* (cellular cytoskeleton).

Among the genes associated with cytokine signaling, the upregulated expression of *IL21R* (Figure 5C,D) demonstrates the readiness of CD8^+^ T cells to respond to IL-21, which supports their cytotoxic function and prevents exhaustion [42]. Upregulated *NEAT1* (Figure 5C,D), a long non-coding RNA, is induced during HIV infection and exhibits antiviral activity by promoting nuclear retention of viral transcripts; its knockdown enhances HIV replication [43]. We also noted the increased expression of the *RGS1* gene (Figure 5C,D), which is responsible for T-lymphocyte proliferation and tissue infiltration [44].

Among the downregulated genes, *HLA-C* (Figure 5C,D) is of particular interest because, unlike other MHC-I proteins (HLA-A, HLA-B), the product of this gene is typically resistant to Nef-mediated degradation due to the absence of a specific tyrosine residue [45]; this fact may indicate specific adaptations of Russian HIV-1 variants aimed at immune evasion.

### 3.5. CD8^+^ T-Effector Memory Cells (CD8^+^ Tem)

During the acute phase of HIV infection, effector memory CD8^+^ T cells (CD8^+^ Tem) actively proliferate and express cytotoxic granules to lyse infected cells [46]. However, even at this early stage, their strong pro-inflammatory cytokine profile can cause hyperactivation, potentially leading to premature exhaustion and dysfunction.

**Figure 6:**
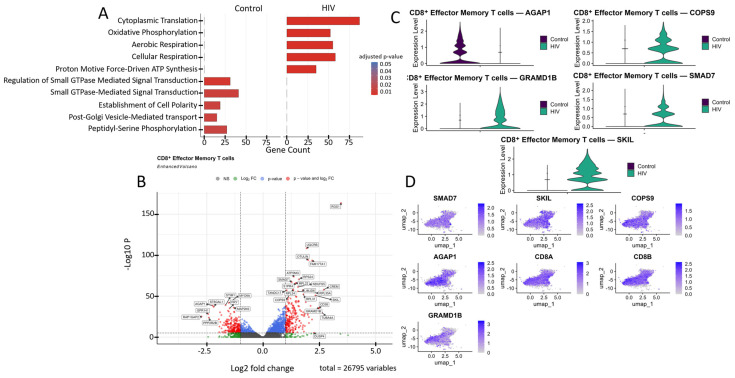
Transcriptomic profile of CD8^+^ Tem cells: (**A**) GO enrichment analysis for control and HIV-positive cohorts. (**B**) A volcano-plot demonstrating highly and lowly expressed genes in the CD8^+^ Tem cells of HIV-positive cohort showing statistically significant differential expression compared to the control cohort. (**C**) Expression levels of key genes across cohorts. (**D**) Expression levels of key genes within the HIV-positive CD8^+^ Tem cell cluster.

Analysis of CD8^+^ Tem cells gene expression in the HIV-positive cohort via GO annotation revealed a predominance of genes involved in metabolic processes, such as cytoplasmic translation and oxidative phosphorylation. In contrast, cells from healthy donors exhibited a predominance of signaling pathways associated with the regulation of cell polarity, protein modification, and GTPase-dependent signal transduction (Figure 6A).

The most significantly (*p* < 0.001) upregulated genes in CD8^+^ Tem cells of HIV-positive patients were (Figure 6B): *RGS1*, *CD38*, *DUSP4*, *S1PR4*, *OTULIN*, *SMAD7*, and *FAM177A1* (regulation of immune response and signaling); *UQCRB*, *ATP5MG*, *NDUFS5*, and *TXNDC17* (ATP generation and other mitochondrial functions); *RPS24*, *RPL22*, *RPL24*, *RPL31*, and *RPL35A* (protein biosynthesis, ribosomal genes); *CREM* and *JMJD6* (transcriptional regulation); *TUBA4A* (microtubule structural component); *GRAMD1B* (cholesterol transport); and *COPS9* (protein processing). Among the downregulated genes in CD8^+^ Tem of HIV-positive individuals were: *STIM1*, *RAP1GAP2*, *PPP2R2B*, *MAP2K6*, and *GPR141* (regulation of intracellular signaling, immune activation, and apoptosis); *MYO9A* (cytoskeleton organization and migration); *ST6GAL1* (protein modification); *AGAP1* (intracellular transport and cytoskeleton remodeling); and *FRY* (cell division and morphogenesis).

Of particular interest is the expression of the cytokine signaling regulators *SMAD7* and *SKIL* (Figure 6C,D). These are suppressors of TGF-β, which is associated with increased density of CXCR3, CCR5, and CCR7 receptors on memory CD4^+^ T cells [34]. Increased expression was also noted for the gene *COPS9*, which is associated with HIV disease progression and is a component of the COP9 signalosome complex [47]. Hyperexpression of components of this signalosome supports HIV-1 replication in infected cells by facilitating virion entry, participating in T-cell activation, and creating a signaling environment conducive to HIV-1 replication [48]. However, not all genes with statistically significant upregulation are directly associated with HIV infection based on available data. For example, the *GRAMD1B* gene encodes the transmembrane protein Aster-B, which functions as a key regulator of lipid homeostasis by mediating non-vesicular transport of cholesterol from the plasma membrane to the endoplasmic reticulum. According to a recent study, *GRAMD1B* also plays a role in modulating autophagy and the accumulation of phosphorylated tau protein, linking its function to the pathogenesis of neurodegenerative diseases [49].

Another downregulated gene of interest is *AGAP1* (Figure 6B,C). It encodes a protein from the Arf GAP family that activates ADP-ribosylation factor (Arf) GTPases, which are key players in membrane trafficking and cytoskeletal dynamics [50]. *AGAP1* is associated with lysosomal transport, which may facilitate the degradation of viral components, such as virion-encapsulated proteins, following viral-cell fusion. This hypothesis is indirectly supported by one study observing increased *AGAP1* expression, along with other membrane trafficking genes, in NK cells [51].

### 3.6. Non-Classical Monocytes (CD14^+^ CD16^++^)

During acute HIV infection, non-classical monocytes (CD14^+^ CD16^++^) enter an activated state. This activation triggers the production of pro-inflammatory cytokines like TNF-α and IL-1β and increases the expression of genes that control migration. These monocytes can then cross the blood–brain barrier via the “Trojan horse” model, contributing to the establishment of viral reservoirs in the central nervous system [52].

**Figure 7:**
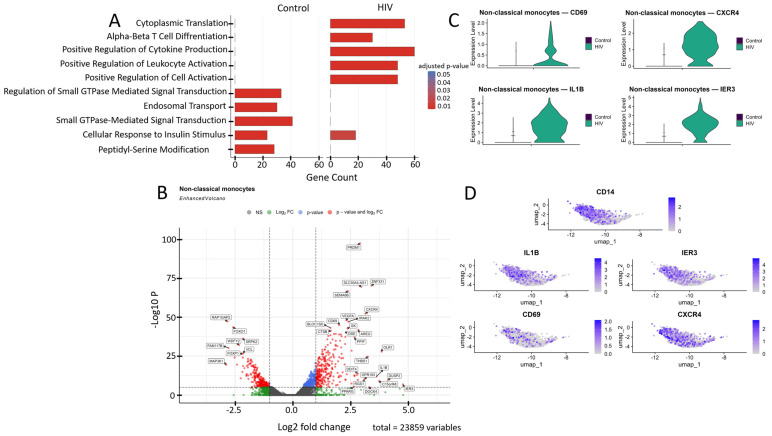
Transcriptomic profile of non-classical monocytes (CD14^+^ CD16^++^): (**A**) GO enrichment analysis for control and HIV-positive cohorts. (**B**) A volcano-plot demonstrating highly and lowly expressed genes in the non-classical monocytes of HIV-positive cohort showing statistically significant differential expression compared to the control cohort. (**C**) Expression levels of key genes across cohorts. (**D**) Expression levels of key genes within the HIV-positive non-classical monocyte cluster.

The transcriptional profile of non-classical monocytes was significantly reprogrammed during acute HIV infection compared to healthy controls (Figure 7A). In HIV-negative donors, pathways related to basic cellular homeostasis are dominant, including the regulation of small GTPases, endosomal transport, and response to insulin stimulus, reflecting their role in maintaining metabolic balance and intracellular trafficking. In contrast, HIV infection triggers a sharp shift toward an immune-activation phenotype, characterized by significant enrichment of pathways for positive regulation of lymphocyte activation, cytokine production, and leukocyte cell–cell adhesion.

Non-classical monocytes demonstrated statistically significant upregulation of the following genes in HIV-positive individuals compared to the control cohort (Figure 7B): *IL1B*, *IRAK2*, *IER3*, *DUSP2*, *AREG*, and *RGS1* (inflammatory response and cytokine signaling); *CXCR4* and *GPR183* (migration, chemotaxis and cellular homing); *DDIT4*, *THBS1*, *PPIF*, *OLR1*, and *C15orf48* (signaling pathways associated with apoptosis, cellular stress and hypoxia response); *PRDM1* and *CD69* (cell activation and differentiation); *VEGFA* (angiogenesis, association with Kaposi’s sarcoma and nephropathy); *GK* and *PPARG* (metabolism); *BLOC1S6* and *DOCK4* (intracellular trafficking and organelle organization); *DSE* and *SEMA6B* (synthesis of extracellular matrix components and adhesion); *CTSB* (protein degradation in lysosomes); and *ZNF331* and *SLC30A4-AS1* (regulation of transcription and RNA splicing). Volcano plot showed several genes with reduced expression in this cell population (Figure 7B), including: *RAP1GAP2*, *MAP3K1*, and *VCL* (regulation of intracellular signaling); *FOXO1*, *FOXP1*, and *FAM177B* (transcription regulation); *WDFY2* (intracellular transport and insulin signaling); and *SPRL2* (unknown function).

Transcriptomic analysis revealed a significant upregulation of genes *CD69*, *CXCR4*, *IL1B*, and *IER3* in non-classical monocytes (Figure 7B,C). This mRNA profile reflects their activated state and enhanced pro-inflammatory potential. CD69 serves as an early activation marker, CXCR4 mediates chemotaxis and migratory activity while potentially facilitating HIV entry, and IL1B and IER3 participate in regulating the NF-κB-mediated inflammatory response and resistance to apoptosis [53].

### 3.7. Natural Killer Cells (NK-Cells)

NK cells actively interact with other immune cells such as dendritic cells (DCs), CD4^+^ T lymphocytes, and CD8^+^ T lymphocytes through direct cellular interactions and cytokine secretion (e.g., TNF-α, IFN-γ) during viral infections. However, in HIV infection, the crosstalk between NK cells and DCs can be impaired, which negatively impacts the functionality of both B and T lymphocytes [54]. The primary function of NK cells is cytotoxicity, mediated by the expression of genes such as *PRF1*, *GZMB*, *CCL3*, and *CCL4* [14].

**Figure 8:**
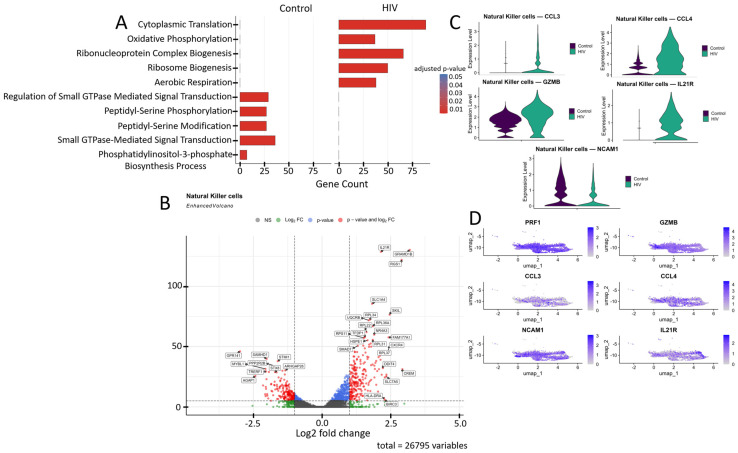
Transcriptomic profile of NK cells: (**A**) GO enrichment analysis for control and HIV-positive cohorts. (**B**) A volcano-plot demonstrating highly and lowly expressed genes in the NK-cells of HIV-positive cohort showing statistically significant differential expression compared to the control cohort.(**C**) Expression levels of key genes across cohorts; (**D**) Expression levels of key genes within the HIV-positive NK cell cluster.

The transcriptomic profile of NK cells shifted from cytoskeletal regulation and signaling toward energy metabolism during acute HIV infection (Figure 8A). In HIV-negative individuals, the dominant pathways include GTPase-dependent signaling, peptidyl-serine phosphorylation, and actin filament organization, which support migration and cytotoxicity mechanisms. In contrast, HIV infection leads to activation of oxidative phosphorylation, aerobic respiration, and mitochondrial ATP synthesis pathways, accompanied by enhanced purine nucleotide biosynthesis.

NK cells from HIV-positive individuals showed upregulated expression of several genes (Figure 8B) including: *IL21R*, *RGS1*, *CXCR4*, *NR4A3*, and *BIRC3* (T-cell activation and signaling); *SLC7A5*, *SLC1A4*, *UQCRB*, *GRAMD1B*, and *HSPE1* (metabolism, amino acid and cholesterol transport, and protein folding); *RPL22*, *RPL31*, *RPL34*, *RPL36A*, *RPL37*, and *RPS11* (ribosomal proteins); *SKIL* and *SMAD7* (TGF-β signaling pathway regulation); *FAM177A1*, *CREM*, and *TFDP1* (transcriptional and epigenetic regulation); *HLA-DRA* (antigen presentation via MHC-II); and *DDIT4* (apoptosis regulation and mTOR signaling inhibition). Several genes in this study group showed downregulated expression compared to controls (Figure 8B): *STIM1*, *PPP2R2B*, and *GPR141* (intracellular signaling and activation regulation); *STX8*, *AGAP1*, and *ARHGAP26* (intracellular signaling, transport, and cytoskeletal dynamics modulation); *SAMHD1* (nucleotide metabolism and HIV-1 replication inhibition); and *TRERF1* (transcriptional regulation and cell cycle control).

The increased expression of *CCL3*, *CCL4*, *GZMB*, and *IL21R* in NK cells (Figure 8B,C) of HIV-positive patients reflects their activated state, characterized by enhanced chemotactic activity (CCL3, CCL4), heightened cytotoxic potential (GZMB), and increased responsiveness to immunomodulatory signals (IL21R). Simultaneously, the reduced expression of *NCAM1* suggests potential impairments in cellular migration and adhesion processes, which may negatively impact NK cell functionality.

### 3.8. Reprogramming of Signaling Cascades in Mononuclear Cells During the Acute Stage of HIV Infection

**Figure 9:**
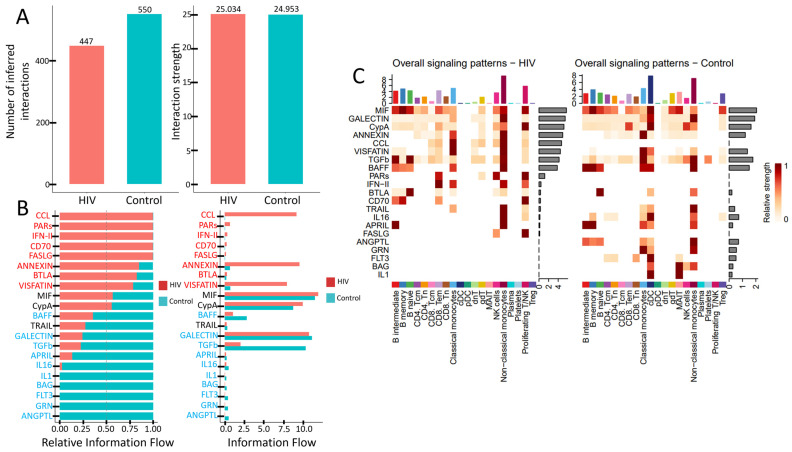
Altered signaling in PBMCs during acute HIV infection: (**A**) Total number and strength of interactions across signaling pathways. (**B**) Activity levels of key signaling pathways. (**C**) Number of active signaling patterns per PBMC subset in HIV-positive (left) and HIV-negative (right) individuals.

HIV infection induces significant rewiring of intercellular communication. These alterations are reflected in the total number of active signaling pathways (Figure 8A), the activity levels of key pathways (Figure 8B), and the signaling diversity across PBMC subpopulations (Figure 9C).

PBMCs from individuals with acute HIV infection showed a reduced number of active signaling pathways, although the interaction strength within the remaining pathways—measured by ligand-receptor pair abundance—was preserved (Figure 9A). Qualitative differences are also evident (Figure 9B). Specifically, signaling pathways such as CCL, PARs, IFN-II, CD70, FASLG, ANNEXIN, BTLA, and VISFATIN showed higher activity in HIV-positive subjects, whereas cascades including BAFF, TRAIL, GALECTIN, TGFb, APRIL, IL16, IL1, BAG, FLT3, GRN, and ANGPTL were more prominent in the control cohort. Notably, pathways like CCL (leukocyte chemotaxis), PARs (cellular response to inflammation), IFN-II (macrophage activation, antiviral response), CD70 (T- and B-cell proliferation and differentiation), and FASLG (apoptosis induction) were nearly inactive in HIV-negative individuals. Conversely, pathways such as IL1 (pro-inflammatory function), BAG (regulation of apoptosis and autophagy), FLT3 (dendritic cell proliferation and differentiation), GRN (tissue inflammation regulation), and ANGPTL (lipid metabolism) showed no detectable activity in HIV-positive individuals.

The number of signaling pathways engaged by individual PBMCs populations also undergoes changes (Figure 9C). CD4^+^ T cells (both CD4^+^ Tcm and CD4^+^ Tem) show reduced interaction strength in the MIF and ANNEXIN pathways, while the IL16 and FLT3 pathways become completely inactive. CD8^+^ T cells decrease their activity in the CypA pathway but engage more actively in the PARs, IFN-II, BTLA, and CD70 pathways. The most pronounced reduction in signaling activity is observed in conventional dendritic cells (cDCs), which lose activity across multiple signaling cascades. In contrast, non-classical monocytes maintain activity in a wide range of pathways. However, they cease signaling via IL1, GRN, and ANGPTL pathways while acquiring activity in the CCL cascade and simultaneously increasing interaction strength in other previously active pathways.

**Figure 10:**
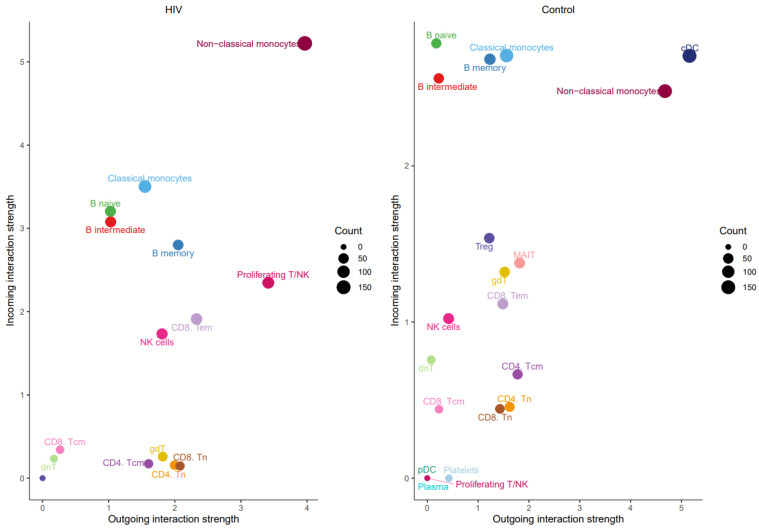
Number of outgoing (*x*-axis) and incoming (*y*-axis) signals for PBMC populations from HIV-positive (**left**) and HIV-negative (**right**) individuals.

The prominent role of non-classical monocytes in intercellular communication was further supported by analyzing the number of incoming and outgoing signals per population (Figure 10).

**Figure 11:**
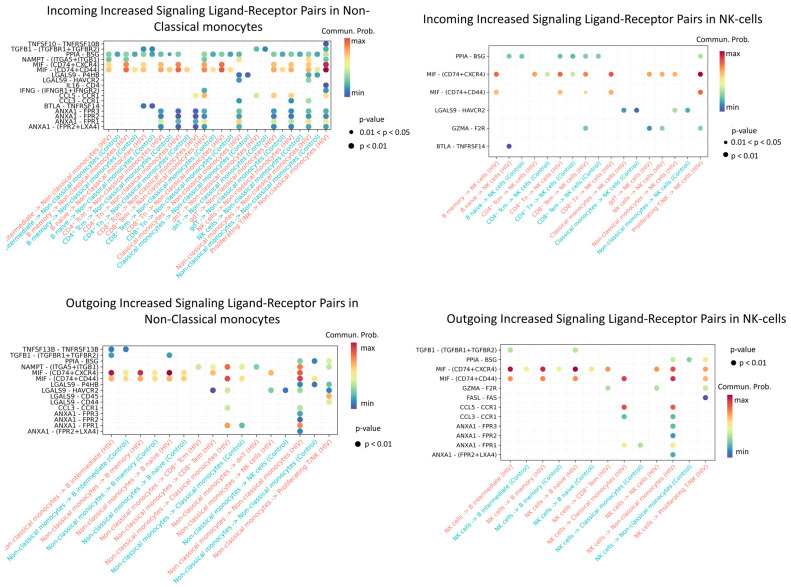
Enriched ligand–receptor interactions in non-classical monocytes and NK cells. Interactions showing higher predicted activity in the HIV-positive cohort compared to controls. The color of the dots indicates the probability of interaction (blue is the minimum probability; red is the maximum probability). If there is no probability of interaction between populations, then it is not displayed on the graph.

Non-classical monocytes emerge as the most active cells in both sending and receiving signals. Increased signaling activity was also demonstrated by NK cells (Figure 11).

Enriched incoming interactions in non-classical monocytes, such as MIF − (CD74 + CXCR4), MIF − (CD74 + CD44), ANXA1 − FPR1, and CCL5 − CCR1, suggest enhanced chemotaxis, adhesion, and pro-inflammatory activation during acute HIV infection. Concurrently, enriched outgoing interactions (NAMPT − (ITGA5 + ITGB); LGALS9 − CD45) reflect increased secretion of factors that modulate intercellular contacts and receptor sensitivity (Figure 11). Together, these changes may contribute to sustaining the inflammatory response during early HIV infection.

Enhanced incoming interactions (MIF − (CD74 + CXCR4; MIF − (CD74 + CD44))) in NK-cells during acute HIV infection indicate heightened susceptibility to MIF-mediated signaling, potentially influencing their activation and targeted migration. Concurrently, increased outgoing interactions, namely PPIA − BSG, MIF − (CD74 + CXCR4/CD44), CCL5 − CCR1, and ANXA1−FPR1, demonstrate expanded secretory capacity of NK cells, which may amplify intercellular communication networks and sustain early inflammatory responses in HIV infection.

**Figure 12:**
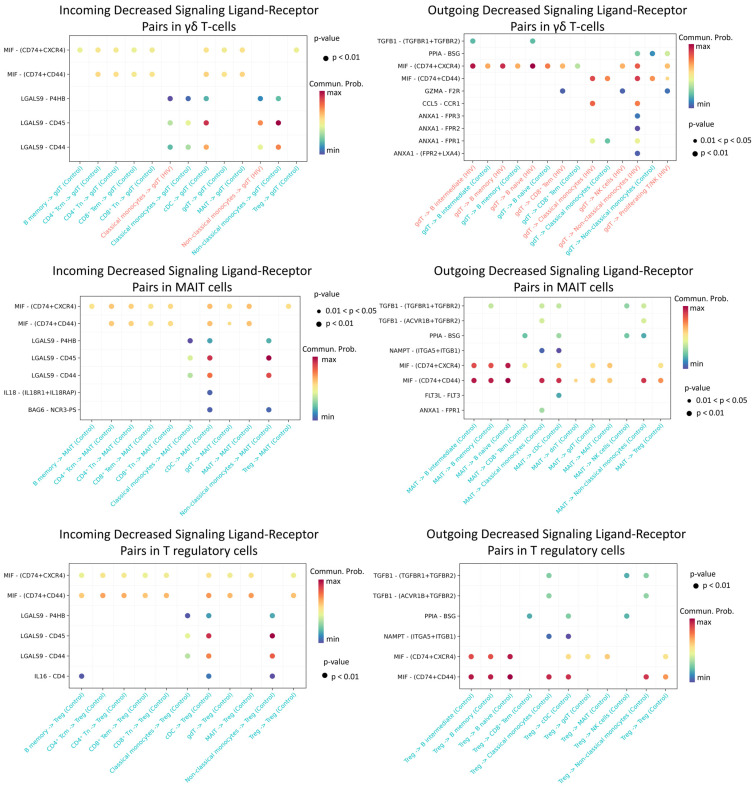
Diminished ligand–receptor interactions in γδ T, MAIT, and T regulatory cells. Interactions showing lower predicted activity in the HIV-positive cohort compared to controls. The color of the dots indicates the probability of interaction (blue is the minimum probability, red is the maximum probability). If there is no probability of interaction between populations, then it is not displayed on the graph.

The most significant decline in activity was recorded for Treg, MAIT, and γδ T cell populations (Figure 12).

We found that γδ T lymphocytes, MAIT cells, and regulatory T cells in HIV-positive individuals exhibit significantly reduced predicted signaling activity through the MIF − (CD74 + CXCR4), MIF − (CD74 + CD44), LGALS9 − CD45, and LGALS9 − CD44 ligand-receptor axes compared to HIV-negative donors. In a normal immune response, MIF binds to CD74 (often complexed with CXCR4 or CD44), promoting inflammatory and anti-apoptotic processes that recruit leukocytes and support their survival [55]. Similarly, galectin-9 (LGALS9) enhances the stability and suppressive function of induced Treg cells via CD44 [56]. During acute HIV infection, γδ and MAIT cells typically mount rapid antiviral responses (e.g., IFN-γ production and cytolytic activity) [57], while Treg cells help restrain excessive mononuclear cell activation. Diminished MIF/Gal-9 signaling likely deprives these cells of crucial activating and chemotactic cues, thereby impairing their efficacy—for instance, blocking Gal-9–CD44 interactions compromises Treg functionality [56]. Collectively, weakened signaling support for γδ, MAIT, and Treg cells during acute HIV infection likely undermines their antiviral capacity and disrupts immune response regulation.

The relative activity level (Figure 8) demonstrates that cytokine signaling pathways (IFN-II, TNF, CD70, VEGF, IL1, IL2, etc.) dominate in the HIV-positive cohort. The absolute, non-normalized activity level of signaling pathways (Information Flow) reveals that these cytokine pathways are practically inactive in the control cohort, while in the HIV-positive cohort they show activity, though lower than other signaling pathways (VISFATIN, CCL, ANNEXIN, MIF; Figure 8).

### 3.9. Intercellular Communication of PBMC During the Acute Stage of HIV Infection

**Figure 13:**
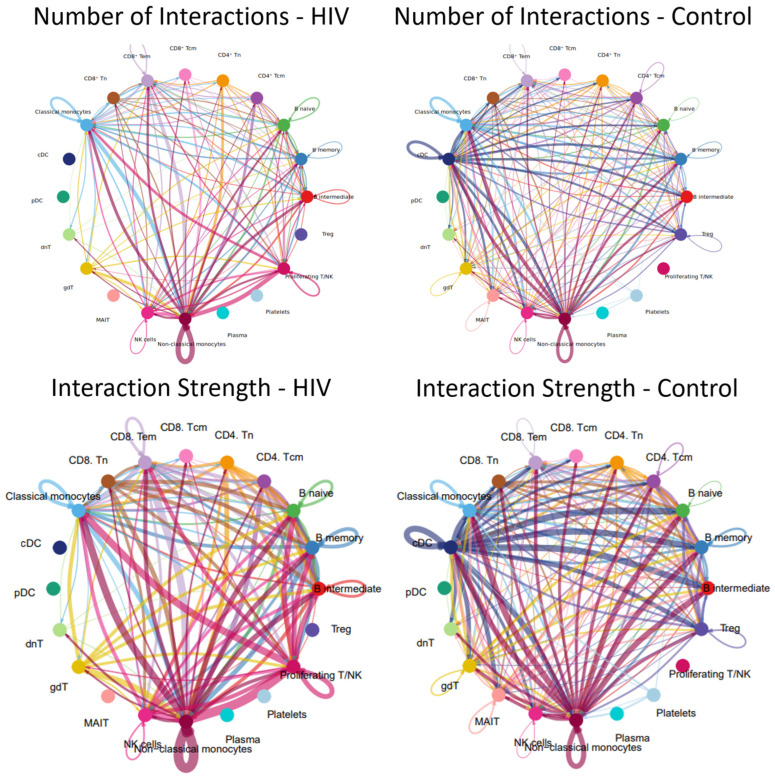
Intercellular communication network in PBMCs. Number and strength of interactions between PBMC populations in individuals with acute HIV-1 infection (**left**) and HIV-negative controls (**right**). Individual colors are assigned to each population and its interactions (lines) with other populations. The thickness of the lines indicates the number of interactions (the number of signaling pathways) between cell populations or the strength of the interactions (the number of ligand-receptor pairs) within the signaling pathways between individual populations. A line running from one population (e.g., nonclassical monocytes) to another population (e.g., classical monocytes) indicates that these populations interact with each other.

Alterations in signaling pathways resulted in changes to the intercellular interaction network (Figure 13).

The results demonstrate that the acute stage of HIV infection is characterized by a substantial reduction in the number and intensity of cellular interactions within PBMC (Figure 13). For instance, dendritic cell populations (cDCs, pDCs) showed minimal communication with other cell types; similarly, Tregs, MAIT cells, and plasma cells were largely disconnected from the network.

Populations exhibiting a significant reduction—though not a complete loss—of intercellular communication included CD4^+^ T cells (Tcm, Tem), CD8^+^ T cells (Tcm, Tem), γδ T cells, and double-negative T (dnT) cells. Relative preservation of numerous interactions is observed in classical (CD14^++^ CD16^−^) and non-classical (CD14^+^ CD16^++^) monocyte populations, consistent with the signaling pathway activity and incoming/outgoing signal analysis where non-classical monocytes similarly emerge as the most active cells.

A more detailed analysis reveals that plasma cells in HIV-positive individuals exhibit significantly fewer interactions with their T-helper cells (Tfh-like and CD4^+^ Tcm cells), whereas these interactions are pronounced in controls. Furthermore, we observed a reduced number of interactions between various T-cell subpopulations (e.g., CD8^+^ Tcm-CD8^+^ Tem, CD4^+^ − CD8^+^ crosstalk, interactions with γδT and dnT cells), whereas this network was more extensive in the control group. Although classical monocytes maintained more contacts than most populations, they lost their central hub-like role in the network, which was instead assumed by non-classical monocytes.

## 4. Discussion

The acute A6/CRF63_02A6 infection was associated with a profound rewiring of PBMC gene expression. We observed a transcriptional signature consistent with robust pro-inflammatory activation, mirroring findings from similar studies, which included pan-activation of NF-κB/TNF and interferon (I/II) pathways and elevated expression of chemokine receptors (e.g., CXCR4) and migration-associated genes. This aligns with prior single-cell studies of hyperacute HIV, which similarly found pervasive interferon-stimulated gene expression and robust cytotoxic/NK cell activation [14]. Consistently, our data showed high expression of granzyme-coding genes (*GZMB*, *GZMH*) and ribosomal genes in memory CD8^+^ T cells and NK cells, indicative of a vigorous antiviral effector response. We also noted elevated metabolic programs, including oxidative phosphorylation components (OXPHOS), across lymphocytes. This metabolic remodeling mirrors recent findings showing that HIV-infected CD4^+^ T cells in a tonsil explant model upregulate OXPHOS genes [58]. Concurrently, transcripts linked to cellular stress and immune activation (e.g., *NR4A* family, *ATF3*, *IER3*) were co-expressed with regulatory or inhibitory factors, suggesting parallel pro- and anti-inflammatory processes.

Key antiviral subsets exhibited both expected and novel patterns. Cytotoxic CD8^+^ T cells and NK cells displayed strong activation signatures but already showed signs of early dysfunction. Specifically, we found a sharp reduction in inferred ligand–receptor interactions between these cytotoxic subsets and other PBMCs, particularly antigen-presenting cells and CD4^+^ T cells. Such functional compartmentalization—reminiscent of the decoupling observed in chronic HIV exhaustion—has not been reported this early in infection. Recent exhaustion atlases have identified HIV-specific “exhausted” CD8^+^ T cell clusters bearing KLRG1/TIGIT in chronic infection [59]. Our finding suggests that A6/CRF63 strains may accelerate features of immune exhaustion. Consistent with a highly activated innate response, plasmacytoid dendritic cells shifted toward an IFN-producing phenotype at the expense of antigen-presentation. HIV is known to drive pDC depletion and massive IFNα release during acute infection [58], and we similarly saw overexpression of IFN-related genes in pDCs. B cells in the acute patients showed increased metabolic gene expression but downregulation of BCR signaling components (e.g., *DOCK8*, *PRKCB*), suggesting impaired antigen responsiveness. These imbalances in innate (pDC) and adaptive (B cell) programs may undermine coordinated antiviral immunity and antibody priming. Notably, there were monocytes in our data on upregulated inflammatory genes (*OLR1*, *SERPINB2*, *COPS9* and *IL1B*), reflecting a hyperactivated state. In line with this, a recent single-cell study found that higher IL1B expression in CD14^+^ monocytes during acute infection was associated with a smaller HIV reservoir [13], suggesting that IL1B-producing monocytes can help restrict viral persistence.

Several regulatory pathways were perturbed in ways that may influence pathogenesis. We detected dysregulation of TGF-β and mTOR signaling. For example, negative regulators of TGF-β (*SMAD7*, *SKIL*, *PMEPA1*) were induced. This may represent a host feedback mechanism, as acute HIV infection has been reported to elevate plasma TGF-β levels. Yim et al. showed that TGF-β upregulation during acute infection can increase CCR5 and CXCR3 on memory CD4^+^ T cells, thereby enhancing R5-tropic HIV entry and reservoir seeding [34]. Our observed induction of TGF-β inhibitors could thus reflect a compensatory attempt to limit TGF-β–driven infection. We also found marked activation of the visfatin/NAMPT pathway. Visfatin is a monocyte-derived adipocytokine that has been shown to selectively inhibit R5-tropic HIV infection in vitro [60]. Its upregulation in our data may therefore represent an innate pressure against R5-using viruses (which typically establish early infection), potentially influencing viral tropism and spread. Together, the TGF-β and visfatin findings suggest that A6/CRF63 infection engages both proviral and antiviral host programs in parallel.

Comparisons with other HIV-1 scRNA studies highlight both commonalities and distinctions. The core acute response—interferon signature, T/NK activation, monocyte inflammation—closely matches published acute-infection profiles [14]. For instance, Lee et al. recently reported that HIV-infected CD4^+^ T cells in early infection adopt a Th17/KLF2 transcriptional program with impaired IFN signaling [61]. We likewise saw evidence of CCR7/FOSL2 upregulation (a Th17/migratory profile) in CD4^+^ T cells, and an overall mixed IFN response across cell types, consistent with the idea that HIV blunts IFN in productively infected cells while driving bystander IFN production. Some findings in our cohort appear distinct from those reported for other subtypes. We observed unexpected downregulation of IL-16 and noncanonical WNT pathways in acute infection; these are not typically suppressed in early HIV and may reflect a subtype-specific strategy to limit new cell recruitment. In general, it is well recognized that HIV subtypes differ in pathogenesis—for example, subtype C often shows slower progression than subtype A or D. Whether A6 or CRF63_02A6 intrinsically drive faster exhaustion or unique signaling patterns remains to be determined, but our data suggest certain differences (e.g., earlier cytotoxic cell dysfunction) compared to published data on other strains.

This study has several technical limitations that must be considered when extrapolating the biologically significant findings to broader populations. First, the analysis is based on a small cohort of HIV-positive patients (n = 3), although these individuals shared similar clinical characteristics. Second, the work focuses solely on transcriptomic profiling and does not incorporate orthogonal validation methods such as flow cytometry or qPCR. Third, we used the results of a scRNAseq data from another study as a control. We did not find a significant batch effect caused by this. However, this fact should be kept in mind when reviewing comparative data between the HIV-positive and the control cohort of the study. Finally, we did not compare the transcriptomic data for HIV infection caused by CRF63_02A6 and A6 with cases caused by other HIV-1 strains. Therefore, this work is rather descriptive in nature and contributes to the formation of a global atlas of transcriptomic data of blood cells in HIV infection.

In conclusion, it is worth saying that the conducted research complements the existing single-cell transcriptomic landscape of acute HIV-1 infection by providing the detailed profile associated with the FSU-dominant variants A6 and CRF63_02A6. Our data refine the understanding of early immune dysregulation by characterizing transcriptional patterns in key immune populations, including the pronounced dysregulation of the TGF-β/mTOR axis and the early disruption of cytotoxic cell communication networks. These findings add a crucial geographical and viral genetic dimension to the global atlas of HIV-1 immunopathogenesis and highlight candidate pathways for further investigation.

## 5. Conclusions

This study provides the comprehensive single-cell transcriptomic analysis of PBMCs during acute HIV-1 infection caused by FSU epidemic variants (subtype A6 and CRF63_02A6). The results reveal profound transcriptional reorganization in key immune populations, including CD4^+^ and CD8^+^ T cell subsets, monocyte subsets, NK cells, B cells, and plasmacytoid dendritic cells.

We identified activation of pro-inflammatory pathways (NF-κB, TNF, IFN-I/II), upregulation of migration genes (*CXCR4*, *CCR7*), markers of metabolic adaptation (OXPHOS components), and complex apoptotic regulation. Crucially, we identified specific infection progression marker genes (*CCR5*, *CXCR4*, *NFAT2*, *OLR1*, *IL1B*) and potential control factors (*NAF1*, *NEAT1*, *PIK3CD*), along with disrupted intercellular communication, particularly between cytotoxic cells (NK, CD8^+^ Tem) and antigen-presenting/regulatory populations.

This study complements the existing single-cell transcriptomic landscape of acute HIV-1 infection by providing the first detailed profile associated with the FSU-dominant variants A6 and CRF63_02A6. Our data refine the understanding of early immune dysregulation by characterizing strain-specific transcriptional patterns in key immune populations, including the pronounced dysregulation of the TGF-β/mTOR axis and the early disruption of cytotoxic cell communication networks. These findings add a crucial geographical and viral genetic dimension to the global atlas of HIV-1 immunopathogenesis and highlight candidate pathways for further investigation.

## Data Availability

The gene expression matrices are available in the Zenodo repository (DOI: 10.5281/zenodo.18365860). Code and other processed file formats are available from corresponding authors upon reasonable request.

## Supplementary Materials

The following supporting information can be downloaded at https://www.mdpi.com/article/10.3390/v18020204/s1, Supplementary Figure S1, ‘Validation analysis of pseudobulk differential expression (70% subsampling)’; Supplementary Figure S2, ‘UMAP graph before integration’; Supplementary Figure S3, ‘UMAP graph after integration’; Supplementary Figure S4, ‘UMAP graph for separate donors’; Supplementary Table S1, ‘Clinically relevant information for HIV-positive patients’; Supplementary Table S2 (markers of cell clusters); Supplementary Table S3, ‘Results of the validation pseudobulk differential expression analysis (70% subsampling)’; Supplementary Table S4, ‘The level of gene expression within the study groups’; Supplementary Table S5, ‘Results of pseudobulk RNAseq analysis’.

## Abbreviations

The following abbreviations are used in this manuscript:

Peripheral blood mononuclear cell	PBMC
Single-cell RNA sequencing	scRNAseq
Gene Ontology	GO
CD8^+^ T Effector Memory Cell	CD8^+^ Tem cell
CD8^+^ T Central Memory Cell	CD8^+^ Tcm cell
Antiretroviral Therapy	ART
Central Nervous System	CNS
Circulating Recombinant Form	CRF
People Who Inject Drugs	PWID
People Living With HIV	PLWH
Near Full Length Genome	NFLG
Principal Components	PCs
Shared Nearest Neighbor	SNN
Uniform Manifold Approximation and Projection	UMAP
Mucosal-associated T Cell	MAIT
Gamma-Delta T cell	γδT cell
Double Negative T cell	dnT cell
Unique Recombinant Form	URF
Regulatory T Cell	Treg cell
CD4^+^ Central Memory T cell	CD4^+^ Tcm
People Live With HIV	PLWH
oxidative phosphorylation	OXPHOS
	
