# Supplementary figures and images for: Single-Cell Transcriptomic Profile Associated with Sub-Subtype A6 and CRF63-02A6 HIV-1 Strain Infection
Source: Viruses. 2026 Feb 4;18(2):204. doi: 10.3390/v18020204 (PMC12944885; doi:10.3390/v18020204)

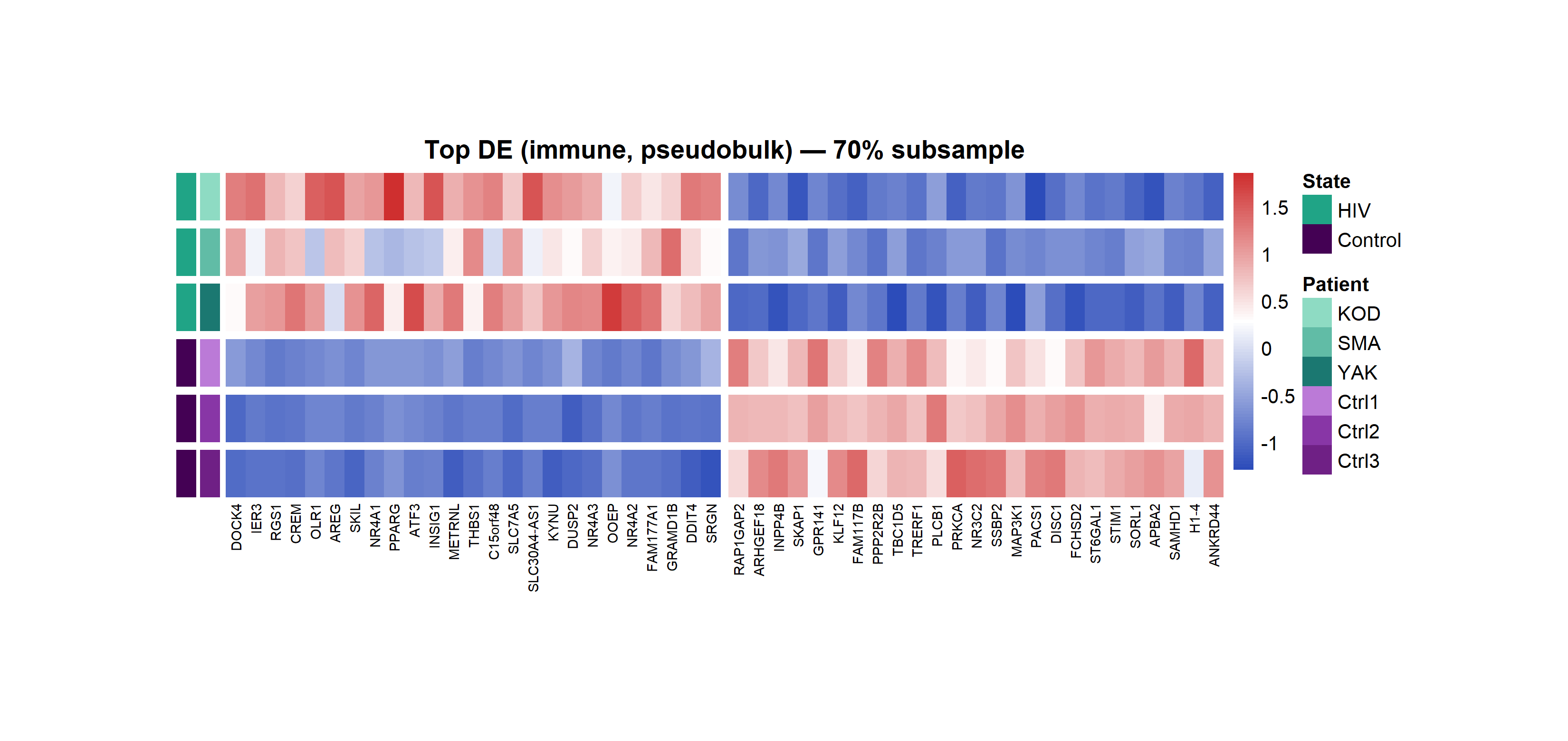

Supplement: Supplementary file 1 [file viruses-18-00204-s001.zip › Supplementary Figure 1 'Validation analysis of pseudobulk differential expression (70% subsampling)'.tiff]

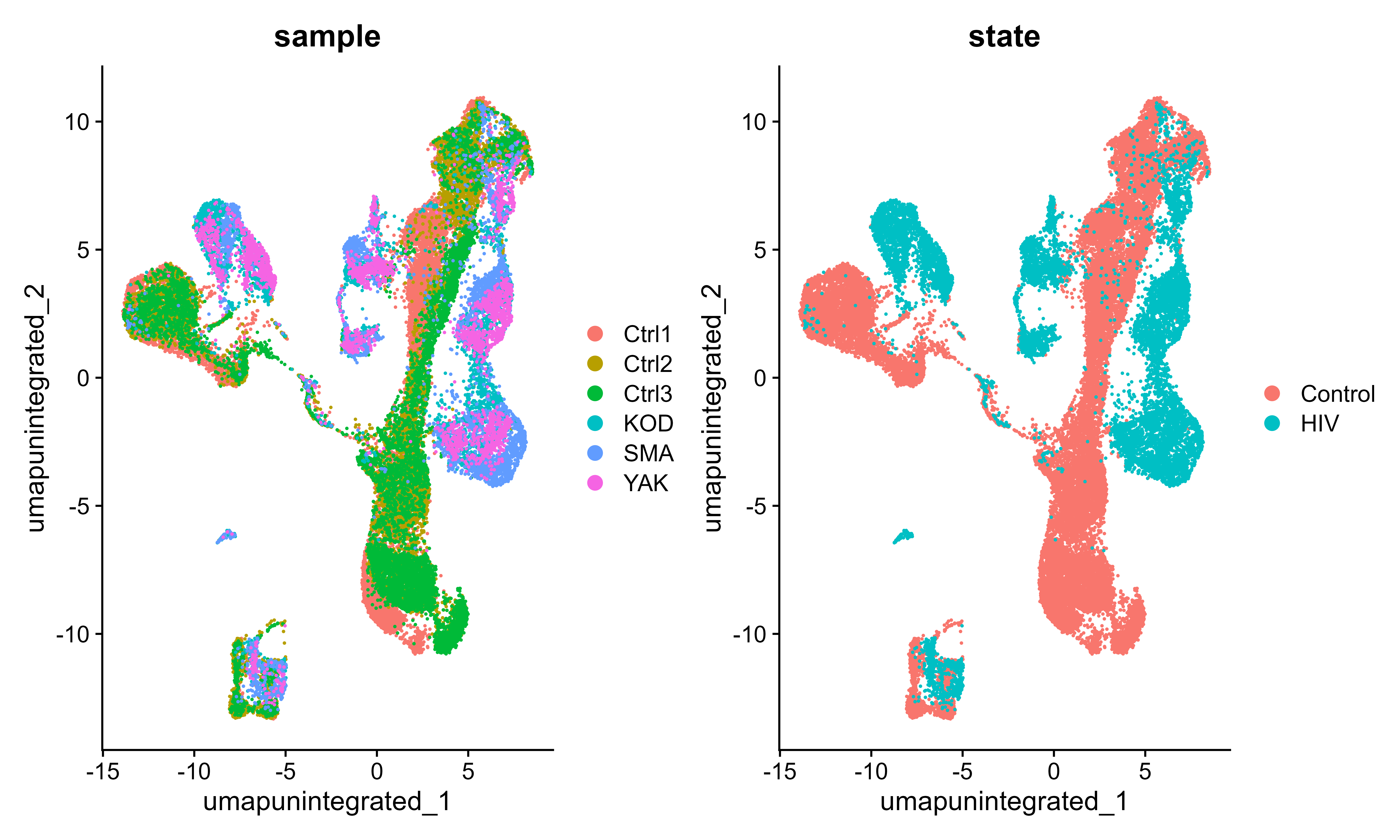

Supplement: Supplementary file 1 [file viruses-18-00204-s001.zip › Supplementary Figure 2 'UMAP graph before integration'.png]

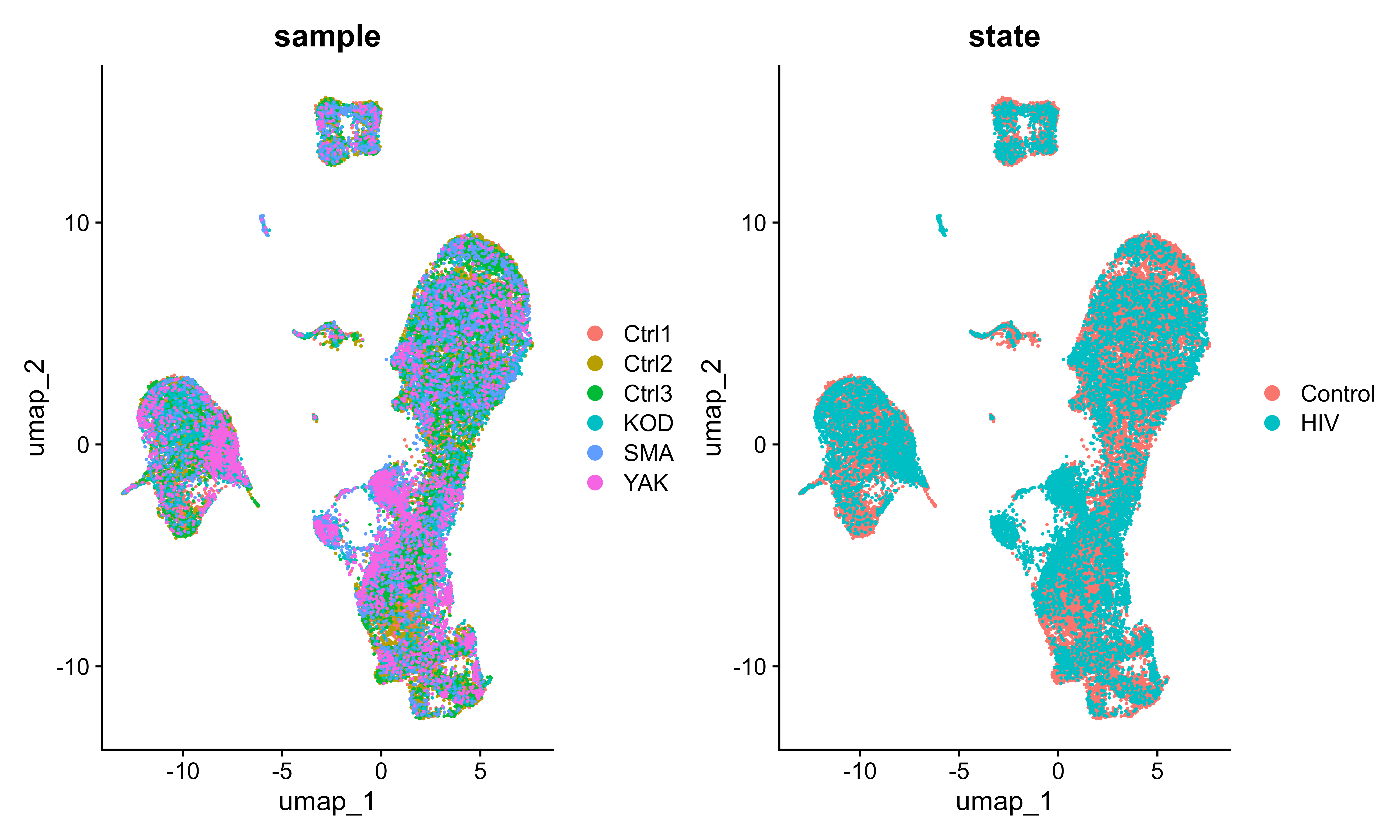

Supplement: Supplementary file 1 [file viruses-18-00204-s001.zip › Supplementary Figure 3 'UMAP graph after integration'.png]
